# Serum ferritin levels, socio-demographic factors and desferrioxamine therapy in multi-transfused thalassemia major patients at a government tertiary care hospital of Karachi, Pakistan

**DOI:** 10.1186/1756-0500-4-287

**Published:** 2011-08-11

**Authors:** Haris Riaz, Talha Riaz, Muhammad Ubaid Khan, Sina Aziz, Faizan Ullah, Anis Rehman, Qandeel Zafar, Abdul Nafey Kazi

**Affiliations:** 1 House officer, Civil Hospital Karachi, Pakistan; 2 Medical graduates, Civil Hospital Karachi, Pakistan; 3 Associate Professor, Department of Paediatrics, Dow University of Health Sciences, Karachi, Pakistan; 4 Electives students, Department of Paediatrics, Civil Hospital Karachi, student of King's College, London, UK; 5 Medical student, Dow Medical College, Karachi, Pakistan

**Keywords:** Beta thalassemia major, Ferritin, Desferrioxamine, Socio-demographic factors, Pakistan

## Abstract

**Background:**

Beta thalassemia is the most frequent genetic disorder of haemoglobin synthesis in Pakistan. Recurrent transfusions lead to iron-overload manifested by increased serum Ferritin levels, for which chelation therapy is required.

**Findings:**

The study was conducted in the Pediatric Emergency unit of Civil Hospital Karachi after ethical approval by the Institutional Review Board of Dow University of Health Sciences. Seventy nine cases of beta thalassemia major were included after a written consent. The care takers were interviewed for the socio-demographic variables and the use of Desferrioxamine therapy, after which a blood sample was drawn to assess the serum Ferritin level. SPSS 15.0 was employed for data entry and analysis.

Of the seventy-nine patients included in the study, 46 (58.2%) were males while 33 (41.8%) were females. The mean age was 10.8 (± 4.5) years with the dominant age group (46.2%) being 10 to 14 years. In 62 (78.8%) cases, the care taker education was below the tenth grade. The mean serum Ferritin level in our study were 4236.5 ng/ml and showed a directly proportional relationship with age. Desferrioxamine was used by patients in 46 (58.2%) cases with monthly house hold income significant factor to the use of therapy.

**Conclusions:**

The mean serum Ferritin levels are approximately ten times higher than the normal recommended levels for normal individuals, with two-fifths of the patients not receiving iron chelation therapy at all. Use of iron chelation therapy and titrating the dose according to the need can significantly lower the iron load reducing the risk of iron-overload related complications leading to a better quality of life and improving survival in Pakistani beta thalassemia major patients.

Conflicts of Interest: None

## Background

Beta thalassemia ranks first amongst the genetic disorders associated with haemoglobin synthesis in terms of prevalence and it is the result of an inherited defect in the synthesis of the beta chain of the adult haemoglobin. Consequently the erythropoiesis is defective and anemia is produced [1]. Considering the 5 to 7% carrier rate in Pakistan, the number of carriers is expected to approach 10 million [2]. This grades thalassemia as one of the most common inherited disorders in Pakistan [3]. Due to lack of appropriate documentation, precise data pertaining to the incidence and prevalence of the disease are lacking. However, different studies statistically estimate that the number of thalassemia major patients born each year is in the order of 4000 to 9000 [4,5]. Due to repeated blood transfusions, all thalassemic patients require Iron Chelation Therapy (ICT) in order to reduce the increased iron load that if not eliminated properly can result in life threatening complications, including severe cardiac toxicity in the second decade of life, producing a significant decrease in the total life expectancy of these patients [6]. However, the practice of repeated parenteral ICT therapy imposes various economic burdens in those who cannot afford such a therapy. Oral chelators such as Deferasirox are much more expensive than the traditional ICT's which makes access difficult for the majority of socio-economically under-privileged patients [7]. Desferoxamine (DFE), a parenteral drug used widely as ICT has been employed in the treatment of beta thalassemia for nearly four decades and has shown to be effective in reducing the hepatic and extra-hepatic adverse affects, particularly myocardial toxicity [8]. However, despite the protracted method of 8- to 10- hours of infusion at least five times a week, the patients suffering from beta thalassemia are often not able to achieve the desired threshold of Ferritin levels in their blood [9]. This study also highlighted the dismal situation of ICT having a negative impact on health related quality of life (HRQOL), and that in most of the cases compliance with ICT being suboptimal, signifying non-satisfactory provision of chelation therapy with respect to economic, clinical and quality of life related outcome.

The aim of the present study is to assess the socio-demographic factors, serum Ferritin levels and adherence to iron chelation therapy in multi-transfused Thalassemia major patients. The series is composed mainly of Thalassemics children and young patients.

### Methodology

The present study is a continuation of the work on Thalassemia major patients and is carried out on the same patients in which the sero-prevalence of viral hepatitis and HIV was determined, published in the Tropical Dr. Journal [10]. However, the objectives are entirely different from the one published.

### Study Design

This is a descriptive cross-sectional study, conducted from July to September 2009 at Karachi, Pakistan's largest city both in terms of area and inhabitants.

### Setting

This study was conducted at Civil Hospital Karachi, one of the largest government tertiary care hospitals of Karachi. The hospital has a Paediatric Emergency Unit (PEU) specialized for the treatment of children presenting with acute medical problems. Patients with transfusion dependent Thalassemia (Thalassemia major) also present to the PEU for the routine follow up and transfusions. The fee structure of the hospital is such that patients with lower socioeconomic strata of the city and those referred from other centres are treated.

### Participants

Diagnosed cases of Thalassemia major that had undergone at least ten transfusions were included after obtaining a written consent from their care takers. Those who did not give consent or had lesser number of transfusions were excluded from the study.

### Study

The sampling technique was non-probability purposive sampling. The care takers of the Thalassemia major patients providing consent were interviewed by the students of Dow Medical College (DMC) through a well structured questionnaire prepared after literature search and modified in terms of questionnaire clarity. The first section comprised of Socio- Demographic variables. These included age, gender, area of residency, status of house (whether owned or at rent), monthly house-hold income and education of the parents. The second part was pertaining to history of vaccination for Hepatitis B and blood group. At the completion of interview a blood sample was obtained by venepuncture from the patient for serological analysis of serum Ferritin levels.

### Ethical Approval

The ethical approval for the study was obtained from the Ethical Review Board (ERB) of the Dow University of Health Sciences (DUHS).

## Results

A total of 79 patients were enrolled in the study duration. The mean age was 10.8 ± 4.5 years. As shown in Table 1, 46 (58.2%) were males where as the rest i.e. 33 (41.8%) were females. Half of the patients were between ages 9 and 15 years.

**Table 1:** Socio-demographic data of Thalassemia patients and their care takers:

Variables		Numbers	Percentage
**Gender**	Male	46	58.2
	
	Female	33	41.8

	**In years**		
	
**Age**	Under 5	13	16.3
	
	5 to 9	18	23.2
	
	10-14	37	46.5
	
	15-above	11	14.0

	A	17	21.6
	
**Blood groups**	B	21	26.4
	
	AB	2	2.6
	
	O	39	49.4

	Il-literate	31	39.4
	
**Education of the care taker**	Primary	31	39.4
	
	Matric	12	15.2
	
	Graduate	5	6.1

**Socioeconomics**	Less than 3000	26	33.3
	
**(monthly house-hold income)**	3000-5000	19	24.2
	
	5000-10,000	29	36.4
	
	10,000-20,000	5	6.1

**Status of house**	Own	60	75.8
	
	On rent	19	24.2

**Vaccination for Hep B**	Done	36	45.5
	
	Not done	33	42.4
	
	Don't remember	10	12.1

Blood group "O" was found to be the predominant. The results highlight the education status of the patients showing that 78.8% of the care takers of the patients had education less than the Matric (39.4% stating that they "never went to school", and an equal number responding that they left the school during the primary education). Most of the patients belonged to a poor socio-economic background reflected from the monthly house hold income with all the care takers having income below 20,000 Pakistani Rupees PKR (Approximately 245 US dollars) per month. The preponderance (36.4%) lies between 5000-10,000 PKR (61 US dollars-122 US dollars) per month. Approximately three fourths (75.8%) stated that they owned a house. The vaccination history revealed that less than half (45.5%) care takers stated that they vaccinated their children for Hepatitis B while 12.1% not even knew whether they have carried out the procedure or not.

**Figure 1:**
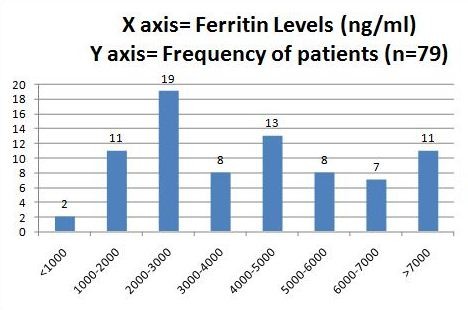
**Serum Ferritin levels (expressed in ng/ml) and frequency of patients in the specified range**.

**Figure 2:**
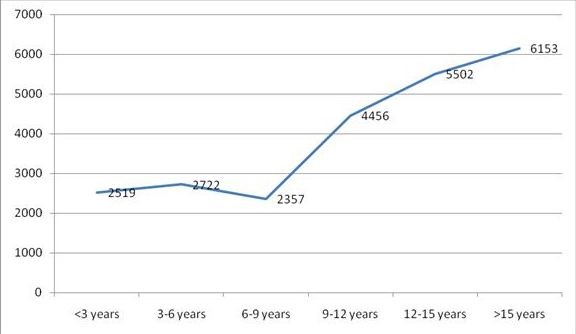
**Relationship between ages (in years) and serum Ferritin Levels (expressed in ng/ml)**.

The mean serum Ferritin levels were found to be 4236.5 (± 2378.3) ng/ml. The values in males were similar (4075.2 ± 2440.7) ng/ml compared to females (4461 ± 2306.6) ng/ml. The figure was less than 1000 ng/ml in just 2 cases, whereas the values were between two to three thousand ng/ml in 19 cases. Figure 1 displays the serum Ferritin ranges and the number of patients who fall within a specified range. 11 cases had serum Ferritin values exceeding 7000 ng/ml. The values increased progressively with age above 9 years as suggested in Figure 2.

Desferrioxamine therapy was used by three fifth of the patients. As expected, serum Ferritin values were much lower (3319.6 ± 1925.8) ng/ml in the group who received the therapy compared to those who did not use the medication (5514.8 ± 2383.0) ng/ml. Monthly house hold income was associated with use of Desferrioxamine therapy, with just one third of patients below income of 3000 Rupees per month taking the medication compared to over two thirds above that income.

## Discussion

Iron overload is an unavoidable complication suffered by thalassemia major patients as a consequence of excessive number of blood transfusions. It is so common that it has been referred to a "second disease" during treatment of first [11]. Serum Ferritin is an easy and in-expensive indirect measurement of iron burden, however, a single measure may not provide reliable indication of iron levels. The new non-invasive methods of measuring iron storage in the body such as MRI or SQUID have greater sensitivity, but they have limited use in developing countries such as Pakistan because of cost and complexity. As excessive iron can lead to organ complications, chelation therapy is employed to lower its levels. Borgna-Pignatti et al reported that a lower Ferritin concentration predicted longer survival, and reduced risk of various complications [12]. Many studies concluded that cirrhosis of liver is associated with increase in serum Ferritin level [13-16].

Patients with lower socio-economic strata visit the government tertiary care hospitals as depicted in our results. All the patient's care takers had a monthly income below 20,000 rupees per month. Considering the average 500 mg vial price of desferrioxamine to be 150-180 rupees (Approximately 5000 rupees per month), it's exceedingly difficult for the care takers to cope with the therapy. These prices do not include infusion devices, the cost of which is covered by the hospital through governmental funding. Table 2 depicts the effect of gender and monthly household income on the use of Desferrioxamine therapy.

**Table 2:** Effect of Gender and House Hold Income on Desferrioxamine Use

Variable		Total Number	Percentage
Gender			

	Male	46	

	On DFE	24	52.2

	Without DFE	22	47.8

			

	Females	33	

	On DFE	22	66.6

	Without DFE	11	33.3

			

House hold income (Rs.)	Less than 3000	20	25.3

	On DFE	8	40.0

	Without DFE	12	60.0

			

	3000-5000	35	44.3

	On DFE	23	65.7

	Without DFE	12	34.3

			

	5000-10000	22	27.8

	On DFE	14	63.6

	Without DFE	8	36.4

			

	10000-20000	2	2.5

	On DFE	1	50.0

	Without DFE	1	50.0

DFE = Desferrioxamine

When not provided with desferrioxamine therapy the patients suffer grave consequences of iron over load, the spectrum of which lies from generalized weakness, weight loss, joint pain, abdominal pain to critical illness such as cirrhosis, hepatoma, diabetes, cardiomyopathy, arthritis, arthropathy, hypopituitarism with hypogonadism and death[17]. The organ most sensitive to chronic iron overload is heart. Chronic iron over load in liver increases chances of cirrhosis and hepatic carcinoma, hepatoma. The death occurs mostly due to liver disease, hepatocellular carcinoma, diabetes or cardiomyopathy[18]. However, T2 weighted Magnetic Resonance Imaging (MRI) studies show that Desferrioxamine is not as cardio-protective as the oral chelator deferiprone as oral deferiprone is more effective than desferrioxamine in removal of myocardial iron [19]. Furthermore, Desferrioxamine therapy is associated with adverse effects and demands associated with administration regimen rendering the compliance sub-optimal. A combination regimen of Desferrioxamine with Deferiprone is shown to be the most rapid way of reducing iron-overload in both liver and heart.

The mean serum Ferritin level in our study was 4236.5 ng/ml, which is markedly higher when compared to normal serum Ferritin levels in children in which the mean serum Ferritin level is considered to be in range of 12-122 ng/ml [20]. The values in our study are higher compared with similar regional and international studies. A study conducted in Islamabad, the federal capital of our country showed the mean serum Ferritin level in patients of beta thalassemia was 3390 ng/ml [21]. Cunningham et al in 2004 reported mean serum Ferritin levels in beta thalassemia patients of North America to be 1696 ng/ml [22]. The difference is largely attributable to the difference in the standards of healthcare between the 2 regions. For instance, the dosage was not adjusted with weight in our group of patients, which could contribute to high Ferritin levels. However, Choudhry VP et al in India reported mean serum Ferritin levels to be 6723 ng/ml [23], even higher than in our study.

Age and chelation therapy are significantly linked with Ferritin levels in our study. This is depicted in the graph (Figure 2) below, which outlines that there is a directly proportional relationship between age inyears and the serum Ferritin levels. Approximately four-fifths of the patients in our study had serum Ferritin values exceeding 2000 ng/ml, which is almost ten times higher than the upper limit of normal. As expected, serum Ferritin values were much lower (3319.6 ± 1925.8) in the group who received the therapy compared to those who did not use the medication (5514.8 ± 2383.0). Monthly house hold income was associated with use of Desferrioxamine therapy, with just one third of patients below income of 3000 Rupees per month taking the medication compared to over two thirds above that income. Interestingly, female gender was associated with increased use of Desferrioxamine therapy.

The importance of chelation therapy can be appreciated from the fact that levels of Ferritin are inversely linked to the survival in Thalassemia major patients. A study reported that the 15 year cardiac disease free survival in patients on iron chelation therapy with Ferritin levels below 2500 ng/ml was 91% compared to under 20% with levels in excess of this value [24].

## Limitations

The height and weight (and consequently the body mass index) of the patients was not obtained. The dose and frequency of Desferrioxamine infusions is not ascertained and is also a limitation.

## Conclusions

The problems of poverty, low education level and inadequate provision of health care are the main stumbling blocks in effective treatment of thalassemic patients of iron over load, the complications of which are the main cause of morbidity and mortality in thalassemia major.

## Recommendations

Use of Depefirone, routine measurements of serum Ferritin levels, adherence to guidelines, awareness amongst the public for prevention of the disease and antenatal diagnosis are some of the methods which can decrease the sufferings of not only the patients, but also of their families.

## Competing interests

The authors declare that they have no competing interests.

## Authors' contributions

HR and TR conceived the study. HR, MUK and TR wrote the manuscript under supervision of SA. FU, QZ, ANK and AR contributed in the study design and data analysis. All the authors read and approved the final manuscript.
